# Crystal structure and Hirshfeld analysis of *trans*-di­iodido­bis­[(methyl­sulfan­yl)benzene-κ*S*]platinum(II)

**DOI:** 10.1107/S2056989023003717

**Published:** 2023-04-28

**Authors:** Annika Schmidt, Isabelle Jourdain, Michael Knorr, Carsten Strohmann

**Affiliations:** a TU Dortmund University, Institute for Inorganic Chemistry, Otto-Hahn-Strasse 6, 44227 Dortmund, Germany; b Institut UTINAM CNRS UMR 6213, Equipe "Matériaux et Surfaces Fonctionnels", Université de Franche-Comté, Faculté des Sciences et des Techniques La Bouloie - 16 Route de Gray, 25030 BESANÇON CEDEX, France; Texas A & M University, USA

**Keywords:** crystal structure, Hirshfeld surface analysis, di­thio­ether

## Abstract

The title complex, [PtI_2_(C_7_H_8_I_2_)_2_], represents a further example of a square-planar Pt^II^–di­thio­ether complex. It crystallizes in the monoclinic space group *P*2_1_/*c*. Additional Hirshfeld analyses indicate a C—H⋯π inter­action along the [010] axis to be the most important packing factor.

## Abstract 2

The title complex represents a further example of a square-planar Pt^II^–di­thio­ether complex. It crystallizes in the monoclinic space group *P*2_1_/*c*. Additional Hirshfeld analyses indicate a C—H⋯π inter­action along the [010] axis to be the most important packing factor.

## 1. Chemical context

Di­thio­ethers are a quite useful class of ligands for various transition-metal complexes and their coordination chemistry is well documented (Murray & Hartley, 1981 ▸). As a result of the soft character of the sulfur center, they preferably bond to soft transition metals like the coinage metals (Cu, Ag, Au), mercury(II), or catalytically active noble metals such as rhodium(I), iridium(I), palladium(II) or platinum(II). Apart from structural aspects (Marangoni *et al.*, 1995 ▸) and the investigation of inversion dynamics occurring at the coordinated sulfur atoms (Abel *et al.*, 1984 ▸), these complexes have been reported to have several applications in homogeneous catalysis (Masdeu-Bulto *et al.*, 2003 ▸; Arrayás & Carretero, 2011 ▸). They can form inter­esting luminescent cluster-like structures (Knorr *et al.*, 2014 ▸; Peindy *et al.*, 2007 ▸) and even coordination polymers by coordination to Cu^I^ and Ag^I^ (Raghuvanshi *et al.*, 2017 ▸; Awaleh *et al.*, 2006 ▸). Depending on the metal coordination sphere and the remaining ligands, the preparation of di­thio­ether complexes may yield different isomers. In particular, the isomerism of chalcogenoether complexes with palladium and platinum has been intensively investigated (Vigo *et al.*, 2006 ▸) and the presence of both *trans*- and *cis*-isomers in solution and the solid state were proven. The clarification of the *trans*–*cis* isomerism is therefore of importance.

**Figure 1:**
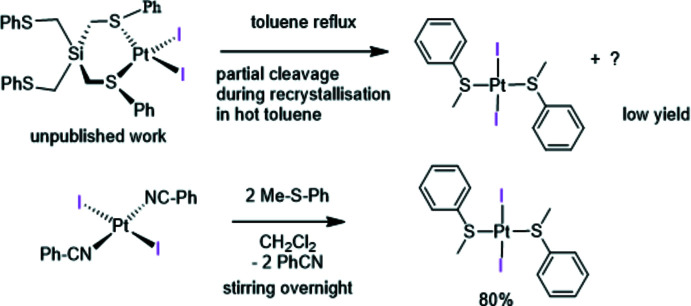
Synthesis scheme for *trans*-PtI_2_(SMePh)_2_] (**1**).

In the past, our groups have investigated the coordination of chelating di­thio­ethers such as the vinylic ferrocenyl-di­thio­ether *Z*-[(ArS)(H)C=C(SAr)-Fc] or the silylated compounds (PhSCH_2_)_2_SiPh_2_ and PhSCH_2_Si(Me)-Si(Me)CH_2_SPh_2_ yielding [Fc-{C(S-*p*-tol­yl)=C(S-*p*-tol­yl)(H)}PtCl_2_], *cis*-[PtCl_2_{(PhSCH_2_)_2_Si_2_Me_4_}] and *cis*-[PtCl_2_{(PhSCH_2_)_2_SiPh_2_}] and converted them *via* metathesis in the presence of NaI to their corresponding di­iodo derivatives (Clement *et al.*, 2007 ▸; Knorr *et al.*, 2004 ▸; Peindy *et al.*, 2006 ▸). We have also shown that the tetra­kis­(thio­ether) (PhSCH_2_)_4_Si can be ligated on HgBr_2_ in a chelating manner (Peindy *et al.*, 2005 ▸). In a similar manner, we also prepared, as shown in Fig. 1 ▸, the complex *cis*-[PtI_2_{(PhSCH_2_)_2_Si(CH_2_SPh}_2_]. When attempting to recrystallize this poorly soluble compound from hot toluene, partial cleavage of the Si—CH_2_Ph bond occurred, yielding *trans*-[PtI_2_(SMePh)_2_] **1**, albeit in a quite low yield of 10%. Alternatively, this air-stable complex could be prepared in a much improved yield of 80% by reaction of bis­(benzo­nitrile)­diiodo­platinum with 2 equivalents of methyl phenyl sulfide (thio­anisol) MeSPh using di­chloro­methane as solvent. This compound was characterized by NMR spectroscopy in solution and exhibits a singlet resonance for the two magnetically equivalent methyl groups at δ 3.01 ppm, flanked by ^195^Pt satellites due to a *^3^J*_PtH_ coupling of 48 Hz. Furthermore, we report herein on the solid-state structure and structural analysis of *trans*-di­iodido­bis­[(methyl­sulfan­yl)benzene-κ*S*]platinum(II) (**1**). In addition, the results of a Hirshfeld analysis of the inter­molecular inter­actions are presented. 
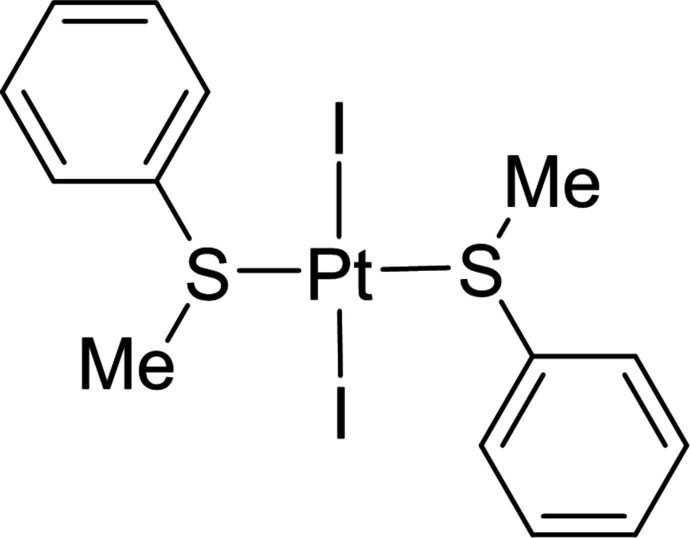


## 2. Structural commentary

**Figure 2:**
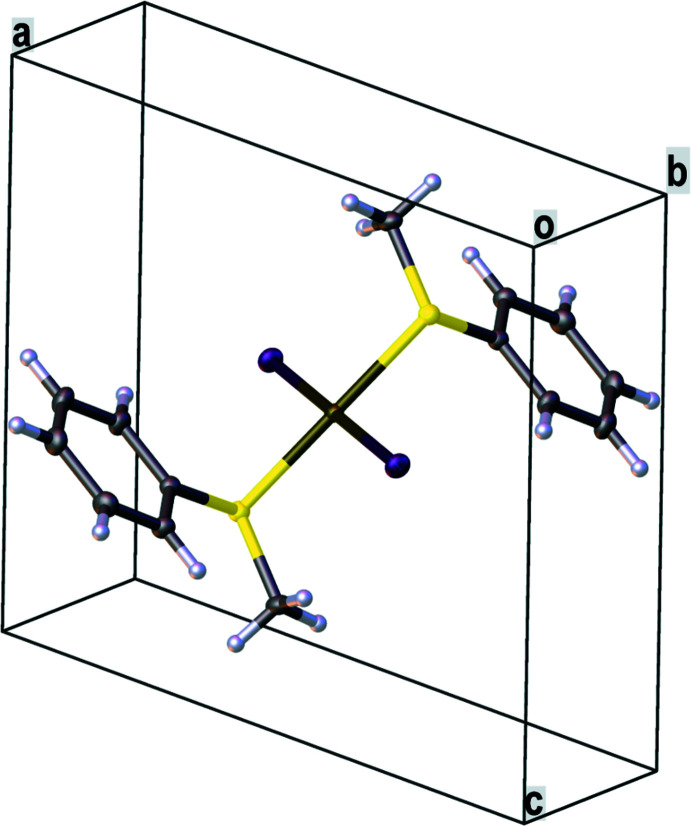
Mol­ecular structure of **1** in the unit cell.

*trans*-Di­iodido­bis­[(methyl­sulfan­yl)benzene-κ*S*]platinum(II) (**1**) crystallizes from di­chloro­methane in the monoclinic crystal system, space group *P*2_1_/*c*. The mol­ecular structure of **1** is presented in Figs. 2 ▸ and 3 ▸ and selected bond lengths and bond angles are given in Table 1 ▸. The asymmetric unit contains half a mol­ecule of **1**, which shows *C*_2*h*_ symmetry**.** The distance from the coordinating iodine center I1 to Pt1 is 2.61205 (15) Å, showing a slight elongation with respect to its educt structure *trans*-[PtI_2_(NCPh)_2_] (**2**) (2.6052 (8) Å; Viola *et al.*, 2018 ▸). The distance from the coordinating sulfur atom S1 to Pt1 is 2.3183 (5) Å. The S1—Pt1 bond is 0.015 Å longer than in the analogous chlorine compound *trans*-[PtCl_2_(SMePh)_2_] (**3**) reported by Ahlgrén (CSD LEQSUW; Vigo *et al.*, 2006 ▸). This elongation may be explained by the thermodynamic *trans*-effect of the opposite halide ligand. Therefore, similar compounds with iodido ligands such as *trans*-[PtI_2_(SMe_2_)_2_] (**4**) (CSD RAYNOU; Lövqvist *et al.*, 1996 ▸) and *trans*-[PtI_2_(tetra­hydro­thio­phene)_2_] (**5**) (CSD SIRPAK; Oskarsson *et al.*, 1990 ▸) show S—Pt bond lengths in the same range at 2.310 (2) and 2.310 (1) Å, respectively. Both complexes also have similar bond lengths for the Pt—I bond [2.6039 (8) Å in **4** and 2.606 (1) Å in **5**]. The chelate complexes *cis*-di­iodo-[1,2-bis(phenyl­sulfan­yl)ethane]­platinum(II) (CSD ZAJWUC; Marangoni *et al.*, 1995 ▸) and *cis*-(1,4-di­thiane-*S*,*S*′)di­iodoplatinum(II) (CSD HUFQAA; Johansson & Engelbrecht, 2001 ▸) are reported to display Pt—I bond lengths of 2.606 (1) and 2.6035 (5) Å, respectively, and somewhat shorter mean Pt—S bond lengths of 2.265 (2) and 2.2751 (16) Å, respectively.

All further bonds have characteristic dimensions (Allen *et al.*, 1987 ▸). The coordination sphere around the platinum center is square-planar. The angles I1—Pt1—I1 and S1—Pt1—S1 are 180°. However, the angle I1—Pt1—S1 of 85.641 (14)° is somewhat more acute. This slight deviation from the ideal angle of 90° is also reported for the chlorido derivative **3** and the dimethyl sulfide analog **4**, as well as in the tetra­hydro­thio­phene analog **5**. The sulfur center shows a distorted tetra­hedral environment with angles C1—S1—Pt1 = 111.00 (8)°, C2—S1—Pt1 = 104.52 (7)° and C2—S1—C1 = 103.46 (11)°.

## 3. Supra­molecular features

**Figure 4:**
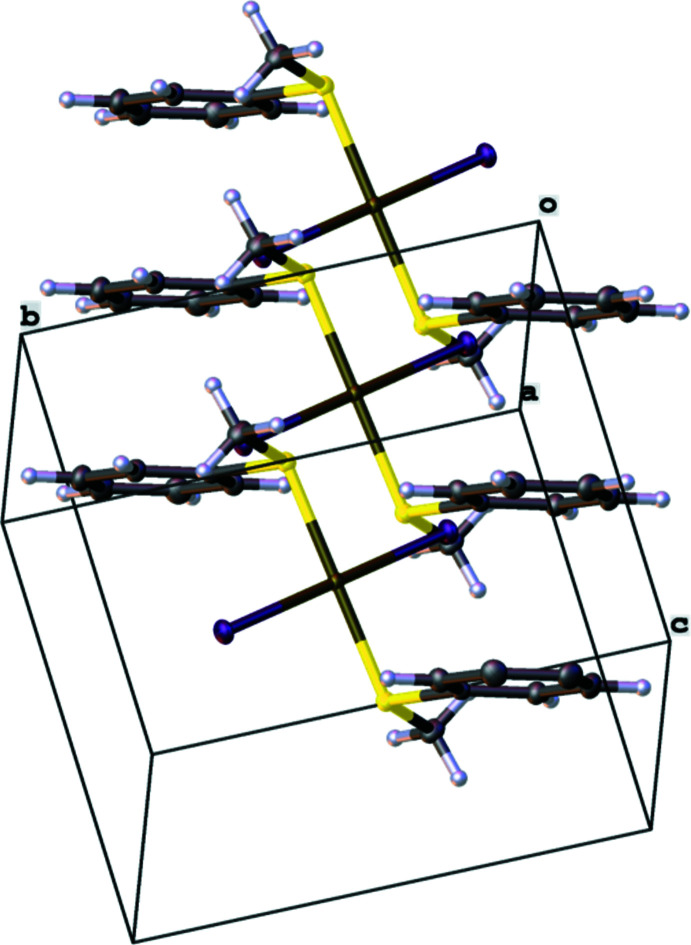
The packing of the solid-state structure of **1** along the [100] axis.

**Figure 5:**
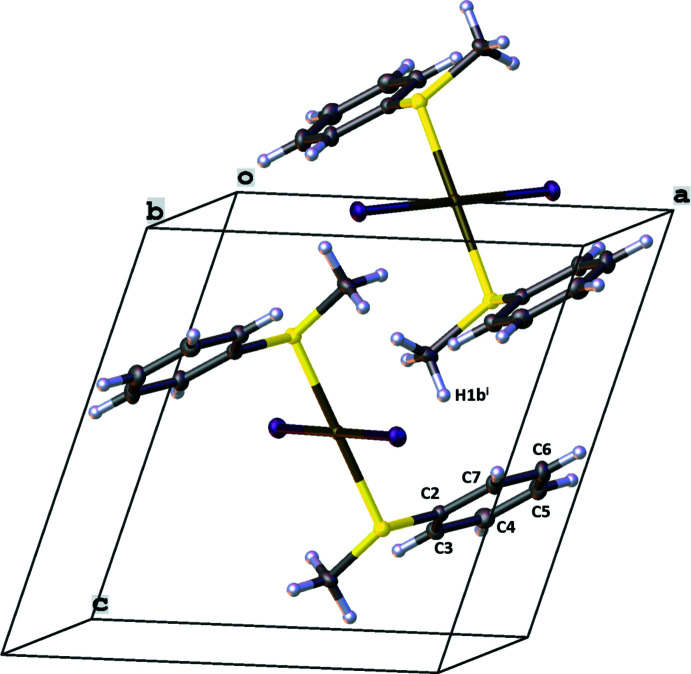
The packing of the solid-state structure of **1** along the [010] axis [symmetry code: (i) *x*, 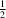 − *y*, − 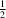 + *z*].

**Figure 6:**
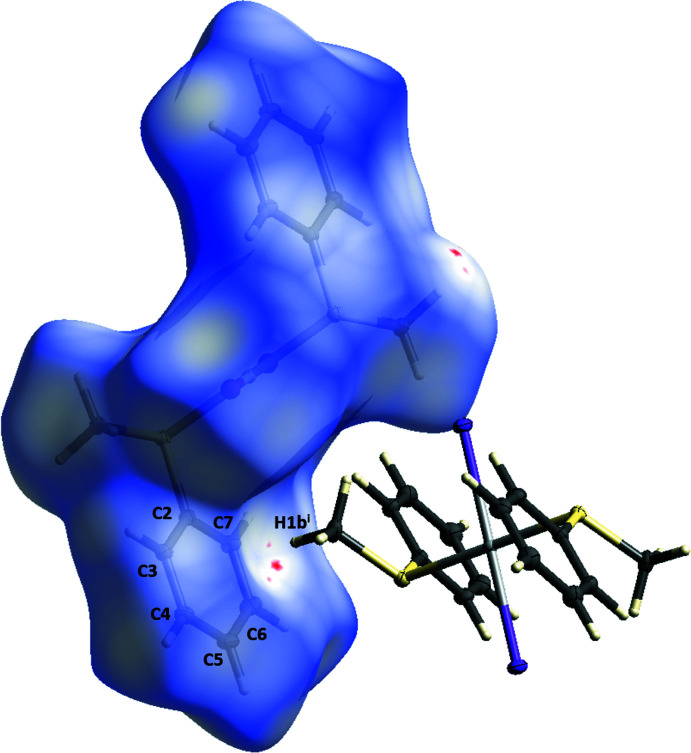
Hirshfeld surface analysis of **1** showing close contacts in the crystal. The π-inter­action between hydrogen atom H1*B* ^i^ and the phenyl ring C2–C7 is indicated by the red spot [symmetry code: (i) *x*, 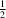 − *y*, − 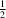 + *z*].

**Figure 7:**
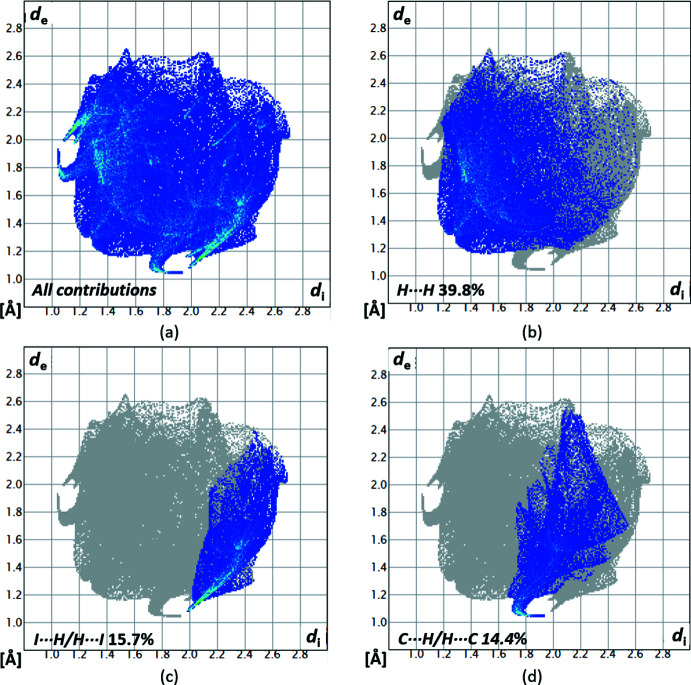
Two-dimensional fingerprint plots for compound **1**, showing (*a*) all contributions, and (*b*)–(*d*) delineated into the contributions of atoms within specific inter­acting pairs (blue areas).

While a repetition of the mol­ecular structure of **1** can be seen along the [100] axis and the [001] axis, as shown in Fig. 4 ▸, the crystal packing along the [010] axis is defined by C—H⋯π inter­actions of the C2–C7 phenyl ring and H1*B* ^i^ [symmetry code: (i) *x*, 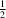 − *y*, − 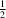 + *z*] with a distance between the phenyl ring and H1*B* ^i^ of 2.5377 (10) Å (Fig. 5 ▸). This inter­action can also be visualized by a Hirshfeld surface analysis (Spackman & Jayatilaka, 2009 ▸) generated by *CrystalExplorer21* (Spackman *et al.*, 2021 ▸). The Hirshfeld surface mapped over *d*_norm_ in the range from −0.0074 to 1.1829 a.u. is shown in Fig. 6 ▸, with the close contact between H1*B* ^i^ and the C2–C7 plane indicated by the red spot. The contributions of the different types of inter­molecular inter­actions for **1** are shown in the two-dimensional fingerprint plots (McKinnon *et al.*, 2007 ▸) in Fig. 7 ▸. The contribution of the H⋯H inter­actions, with a value of 39.8%, has the largest share of the crystal packing of **1**. The remaining hydrogen–heteronuclear inter­actions have a smaller share with a 15.7% contribution for I⋯H, a 14.4% contribution for C⋯H and a 3.6% contribution for S⋯H. The heteronuclear I⋯H and C⋯H inter­actions appear as spikes.

## 4. Database survey

By a search in the Cambridge Crystallographic Database (WebCSD, November 2022; Groom *et al.*, 2016 ▸), various structures of dihalide transition-metal complexes with the same ligand motif as **1** were found. To compare the most similar structures, only dihalide transition metal complexes with the bis­[(methyl­sulfan­yl)benzene] ligand and its oxidized derivative are focused on now. The already compared structure *trans*-di­chloro-bis­[meth­yl(phen­yl)sulfan­yl]platinum (LEQSUW; Vigo *et al.*, 2006 ▸) has been published, as well as its palladium derivative with (SARWEP; Oilunkaniemi *et al.*, 2006 ▸) and without (LEQSOQ; Vigo *et al.*, 2006 ▸) inserted benzene. In addition, *cis*-di­chloro­bis­(methyl­phenyl­sulfoxide)­palladium has been published independently by two different research groups [JISWUD (Antolini *et al.*, 1991 ▸) and JISWUD01 (Gama de Almeida *et al.*, 1992 ▸)]. Further examples of PtI_2_ thio­ether complexes are *cis*-di­iodo-(1,4,7-tri­thia­cyclo­nonane-*S*,*S*′)platinum(II) (ACUXAX; Grant *et al.*, 2001 ▸), di­iodo-(2,9-dimethyl-1,10-phenanthroline)(di­methyl­sulfide)­platinum(II) (BERTIC; Fanizzi *et al.*, 2004 ▸), and di­iodo-(5-phenyl-1-thia-5-phospha­cyclo-octane-*P*,*S*)platinum(II) (KEJHEM; Toto *et al.*, 1990 ▸).

Similar complexes were also structurally characterized by our research groups and include *cis*-[PtBr_2_{(PhSCH_2_)_2_SiPh_2_}] (ECOHAG; Knorr *et al.*, 2004 ▸) and *cis*-[PtI_2_{(PhSCH_2_)_2_SiPh_2_}]·DCM (ECOHIO, Knorr *et al.*, 2004 ▸), which were determined in order to investigate the *trans*-influence of different halide ligands on the Pt—S bond. Further examples of di­thio­ether complexes stemming from our laboratories are *cis*-[PtCl_2_{(PhSCH_2_)_2_Si_2_Me_4_}] (MEDYOK; Peindy *et al.*, 2006 ▸) and *cis*-[PtI_2_{(PhSCH_2_)_2_Si_2_Me_4_}]·DCM (MEDZIF; Peindy *et al.*, 2006 ▸).

## 5. Synthesis and crystallization

*trans*-Di­iodo­bis­[(methyl­sulfan­yl)benzene]­platinum (**1**) was synthesized by adding methyl­phenyl sulfide (37 mg, 0.30 mmol, 1.50 eq.) dissolved in 0.5 mL of di­chloro­methane *via* a microsyringe to a solution of bis­(benzo­nitrile)­diiodo­platinum (65 mg, 0.10 mmol, 1.00 eq.) in di­chloro­methane (3 mL) and stirring overnight at room temperature. *trans*-Di­iodo­bis­[(methyl­sulfan­yl)benzene]­platinum (**1**, 557 mg, 0.80 mmol, 80%) was isolated as red crystals after layering with heptane.

Calculated for C_14_H_16_I_2_PtS_2_ (697.30 g mol^−1^): C, 24.11; H, 2.32; S, 9.20. Found: C, 23.92; H, 2.21; S, 9.05%.

^1^H NMR (400MHz, CDCl_3_): δ = 3.01 (*s*, *^3^J*_PtH_ = 48 Hz, 6H; C*H*_3_), 7.05–7.73 (*m*, 10*H*; phen­yl) ppm.

## 6. Refinement

Crystal data, data collection and structure refinement details are summarized in Table 2 ▸. H atoms were positioned geometrically (C—H = 0.95–1.00 Å) and were refined using a riding model, with *U*_iso_(H) = 1.2*U*_eq_(C) for CH_2_ and CH hydrogen atoms and *U*_iso_ (H) = 1.5*U*_eq_(C) for CH_3_ hydrogen atoms.

## Supplementary Material

Crystal structure: contains datablock(s) I, global. DOI: 10.1107/S2056989023003717/jy2030sup1.cif

Structure factors: contains datablock(s) I. DOI: 10.1107/S2056989023003717/jy2030Isup2.hkl

CCDC reference: 2258409

Additional supporting information:  crystallographic information; 3D view; checkCIF report

## Figures and Tables

**Figure 3:**
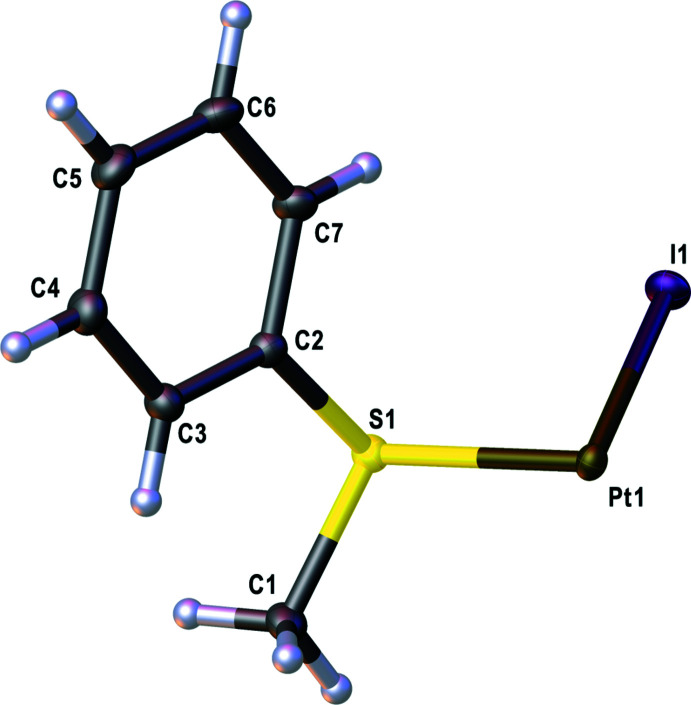
Asymmetric unit of **1** with labeled atoms.

**Table 1:** Selected geometric parameters (Å, °)

Pt1—I1	2.6121 (2)	S1—C2	1.782 (2)
Pt1—S1	2.3183 (5)	S1—C1	1.800 (2)
			
I1^i^—Pt1—I1	180.0	S1—Pt1—I1	85.641 (14)
S1—Pt1—I1^i^	94.359 (14)	C2—S1—C1	103.46 (11)

Symmetry code: (i) 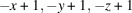 .

**Table 2:** Experimental details

Crystal data	
Chemical formula	[PtI_2_(C_7_H_8_I)_2_]
*M*_r_	697.28
Crystal system, space group	Monoclinic, *P*2_1_/*c*
Temperature (K)	100
*a*, *b*, *c* (Å)	9.5796 (3), 9.5104 (3), 9.7960 (3)
β (°)	107.645 (1)
*V* (Å^3^)	850.48 (5)
*Z*	2
Radiation type	Mo *K*α
μ (mm^−1^)	12.11
Crystal size (mm)	0.31 × 0.25 × 0.20

Data collection	
Diffractometer	Bruker APEXII CCD
Absorption correction	Multi-scan (*SADABS*; Krause *et al.*, 2015 ▸)
*T*_min_, *T*_max_	0.263, 0.498
No. of measured, independent and observed [*I* > 2σ(*I*)] reflections	27554, 4125, 4033
*R*_int_	0.038
(sin θ/λ)_max_ (Å^−1^)	0.833

Refinement	
*R*[*F* ^2^ > 2σ(*F* ^2^)], *wR*(*F* ^2^), *S*	0.021, 0.053, 1.17
No. of reflections	4125
No. of parameters	89
H-atom treatment	H-atom parameters constrained
Δρ_max_, Δρ_min_ (e Å^−3^)	2.26, −1.80

Computer programs: *APEX2* and *SAINT* V8.38A (Bruker, 2018 ▸), *SHELXT2014/5* (Sheldrick, 2015*a* ▸), *SHELXL2019/2* (Sheldrick, 2015*b* ▸), *OLEX2* (Dolomanov *et al.*, 2009 ▸) and *publCIF* (Westrip, 2010 ▸).

## supplementary crystallographic information

### Crystal data

**Table:**

[PtI_2_(C_7_H_8_I)_2_]	*F*(000) = 632
*M**_r_* = 697.28	*D*_x_ = 2.723 Mg m^−^^3^
Monoclinic, *P*2_1_/*c*	Mo *K*α radiation, λ = 0.71073 Å
*a* = 9.5796 (3) Å	Cell parameters from 9775 reflections
*b* = 9.5104 (3) Å	θ = 3.1–36.3°
*c* = 9.7960 (3) Å	µ = 12.11 mm^−^^1^
β = 107.645 (1)°	*T* = 100 K
*V* = 850.48 (5) Å^3^	Prism, red
*Z* = 2	0.31 × 0.25 × 0.20 mm

### Data collection

**Table:**

Bruker APEXII CCD diffractometer	4125 independent reflections
Radiation source: microfocus sealed X-ray tube, Incoatec Iµs	4033 reflections with *I* > 2σ(*I*)
HELIOS mirror optics monochromator	*R*_int_ = 0.038
Detector resolution: 10.4167 pixels mm^-1^	θ_max_ = 36.3°, θ_min_ = 2.2°
ω and φ scans	*h* = −15→15
Absorption correction: multi-scan (SADABS; Krause *et al.*, 2015)	*k* = −15→15
*T*_min_ = 0.263, *T*_max_ = 0.498	*l* = −16→16
27554 measured reflections	

### Refinement

**Table:**

Refinement on *F*^2^	Primary atom site location: dual
Least-squares matrix: full	Hydrogen site location: inferred from neighbouring sites
*R*[*F*^2^ > 2σ(*F*^2^)] = 0.021	H-atom parameters constrained
*wR*(*F*^2^) = 0.053	*w* = 1/[σ^2^(*F*_o_^2^) + (0.0193*P*)^2^ + 1.6484*P*] where *P* = (*F*_o_^2^ + 2*F*_c_^2^)/3
*S* = 1.17	(Δ/σ)_max_ = 0.002
4125 reflections	Δρ_max_ = 2.26 e Å^−^^3^
89 parameters	Δρ_min_ = −1.79 e Å^−^^3^
0 restraints	

### Special details

**Table:**

Geometry. All esds (except the esd in the dihedral angle between two l.s. planes) are estimated using the full covariance matrix. The cell esds are taken into account individually in the estimation of esds in distances, angles and torsion angles; correlations between esds in cell parameters are only used when they are defined by crystal symmetry. An approximate (isotropic) treatment of cell esds is used for estimating esds involving l.s. planes.

### Fractional atomic coordinates and isotropic or equivalent isotropic displacement parameters (Å^2^)

**Table:**

	*x*	*y*	*z*	*U*_iso_*/*U*_eq_	
Pt1	0.500000	0.500000	0.500000	0.01069 (3)	
I1	0.67448 (2)	0.70668 (2)	0.48539 (2)	0.01650 (4)	
S1	0.66656 (6)	0.45396 (6)	0.72234 (6)	0.01359 (8)	
C3	0.7382 (3)	0.1669 (2)	0.7476 (2)	0.0159 (3)	
H3	0.668801	0.154647	0.798352	0.019*	
C7	0.8623 (2)	0.3176 (2)	0.6213 (3)	0.0168 (4)	
H7	0.876638	0.407868	0.586351	0.020*	
C2	0.7595 (2)	0.2989 (2)	0.6957 (2)	0.0134 (3)	
C1	0.5743 (3)	0.3994 (3)	0.8486 (2)	0.0188 (4)	
H1A	0.518020	0.478370	0.868893	0.028*	
H1B	0.646855	0.368646	0.937513	0.028*	
H1C	0.507882	0.321330	0.807968	0.028*	
C4	0.8199 (3)	0.0527 (3)	0.7244 (3)	0.0200 (4)	
H4	0.806058	−0.037609	0.759612	0.024*	
C5	0.9216 (3)	0.0705 (3)	0.6501 (3)	0.0216 (4)	
H5	0.976478	−0.007512	0.633993	0.026*	
C6	0.9426 (3)	0.2037 (3)	0.5991 (3)	0.0209 (4)	
H6	1.012472	0.216130	0.549005	0.025*	

### Atomic displacement parameters (Å^2^)

**Table:**

	*U*^11^	*U*^22^	*U*^33^	*U*^12^	*U*^13^	*U*^23^
Pt1	0.01172 (5)	0.00914 (5)	0.01237 (5)	0.00010 (3)	0.00539 (3)	0.00002 (3)
I1	0.01712 (6)	0.01357 (6)	0.02033 (7)	−0.00397 (4)	0.00794 (5)	0.00006 (4)
S1	0.0149 (2)	0.0124 (2)	0.01360 (19)	−0.00016 (15)	0.00455 (16)	−0.00055 (15)
C3	0.0176 (8)	0.0150 (8)	0.0162 (8)	0.0008 (7)	0.0067 (7)	0.0012 (7)
C7	0.0142 (8)	0.0170 (9)	0.0205 (9)	−0.0018 (7)	0.0073 (7)	−0.0004 (7)
C2	0.0135 (8)	0.0132 (8)	0.0134 (8)	−0.0002 (6)	0.0039 (6)	−0.0010 (6)
C1	0.0226 (10)	0.0214 (10)	0.0147 (8)	0.0047 (8)	0.0092 (7)	0.0018 (7)
C4	0.0229 (10)	0.0164 (9)	0.0206 (10)	0.0038 (7)	0.0063 (8)	0.0029 (7)
C5	0.0194 (10)	0.0210 (10)	0.0244 (11)	0.0073 (8)	0.0065 (8)	−0.0001 (8)
C6	0.0149 (9)	0.0243 (11)	0.0257 (11)	0.0010 (8)	0.0094 (8)	−0.0012 (8)

### Geometric parameters (Å, º)

**Table:**

Pt1—I1	2.6121 (1)	C7—C2	1.403 (3)
Pt1—I1^i^	2.6120 (1)	C7—C6	1.383 (3)
Pt1—S1^i^	2.3183 (5)	C1—H1A	0.9800
Pt1—S1	2.3183 (5)	C1—H1B	0.9800
S1—C2	1.782 (2)	C1—H1C	0.9800
S1—C1	1.800 (2)	C4—H4	0.9500
C3—H3	0.9500	C4—C5	1.392 (4)
C3—C2	1.393 (3)	C5—H5	0.9500
C3—C4	1.397 (3)	C5—C6	1.398 (4)
C7—H7	0.9500	C6—H6	0.9500
			
I1^i^—Pt1—I1	180.0	C7—C2—S1	115.60 (17)
S1^i^—Pt1—I1	94.358 (14)	S1—C1—H1A	109.5
S1—Pt1—I1^i^	94.359 (14)	S1—C1—H1B	109.5
S1—Pt1—I1	85.641 (14)	S1—C1—H1C	109.5
S1^i^—Pt1—I1^i^	85.643 (14)	H1A—C1—H1B	109.5
S1—Pt1—S1^i^	180.0	H1A—C1—H1C	109.5
C2—S1—Pt1	104.52 (7)	H1B—C1—H1C	109.5
C2—S1—C1	103.46 (11)	C3—C4—H4	119.8
C1—S1—Pt1	111.00 (8)	C5—C4—C3	120.4 (2)
C2—C3—H3	120.3	C5—C4—H4	119.8
C2—C3—C4	119.3 (2)	C4—C5—H5	120.1
C4—C3—H3	120.3	C4—C5—C6	119.7 (2)
C2—C7—H7	120.2	C6—C5—H5	120.1
C6—C7—H7	120.2	C7—C6—C5	120.4 (2)
C6—C7—C2	119.6 (2)	C7—C6—H6	119.8
C3—C2—S1	123.88 (17)	C5—C6—H6	119.8
C3—C2—C7	120.5 (2)		
			
Pt1—S1—C2—C3	−107.26 (19)	C1—S1—C2—C7	−168.90 (18)
Pt1—S1—C2—C7	74.82 (17)	C4—C3—C2—S1	−178.09 (18)
C3—C4—C5—C6	0.4 (4)	C4—C3—C2—C7	−0.3 (3)
C2—C3—C4—C5	0.0 (4)	C4—C5—C6—C7	−0.5 (4)
C2—C7—C6—C5	0.2 (4)	C6—C7—C2—S1	178.21 (19)
C1—S1—C2—C3	9.0 (2)	C6—C7—C2—C3	0.2 (3)

Symmetry code: (i) −*x*+1, −*y*+1, −*z*+1.
